# Influence of operating speed, load and mileage on the lubricating performance of self-lubricating rolling linear guide

**DOI:** 10.1371/journal.pone.0328534

**Published:** 2025-07-21

**Authors:** Haiyong Wu, Na Lin, Bihua Shen, Li Liu, Erjun Lu, Guosheng Xu, Qiqin Cai, Yang Chen, Xiaohui Chen

**Affiliations:** 1 School of Equipment Manufacturing and Automobile Engineering, Zhangzhou Institute of Technology, Zhangzhou, Fujian Province, China; 2 Fujian Provincial Higher Education Engineering Center for Applied Technology in Intelligent Manufacturing of Precision Mechanical Functional Components, Zhangzhou Institute of Technology, Zhangzhou, China; 3 Institute of Manufacturing Engineering, Huaqiao University, Xiamen, Fujian Province, China; University of Vigo, SPAIN

## Abstract

Self-lubricating rolling linear guides are widely used in precision instruments, industrial automation production lines, and other fields. The lubrication performance of rolling linear guide has a significant impact on their smooth operation and service life. This paper aims to investigate influence of different operation condition on the lubricating performance of rolling linear guide. Polytetrafluoroethylene (PTFE) is employed as the substrate to fabricate porous media which is installed at the end cover of the rolling linear guide slider to study the effects of operating speed, load, and mileage on the oil film thickness of rolling linear guide. The thickness of lubricating oil film is basically linearly proportional to the operating speed. The influence of load and operating mileage on the thickness of lubricating oil film is not significant. The oil storage rate gradually decreases with the operating mileage increases. The internal pores of porous media have good connectivity. The interconnection of various pores is beneficial for oil storage, oil control, and oil leakage during the operation of rolling linear guides. The self-lubricating rolling linear guide is in a fully film elastohydrodynamic lubrication state which can achieve good lubrication for the rolling linear guide. The results of the studies could provide theoretical reference for improving the self-lubricating performance of rolling linear guides.

## 1. Introduction

Self-lubricating rolling linear guide is a high value-added, maintenance free rolling linear guide that can be used in environments with high cleanliness and inconvenient lubrication for high-altitude operations [1,2], such as semiconductor equipment (precision lithography machines, etc.), food and drug equipment (vacuum packaging automation lines, etc.), high-end medical equipment (CT machines, etc.), as well as aerospace equipment (aviation optical remote sensing mechanisms, image scanning mechanisms, etc.), wind power generation, and other fields [3,4].

The self-lubricating of rolling linear guides is basically achieved by using self-lubricating units installed in the slider raceway or end cover [5–7]. The friction pair interface of rolling linear guide is covered with lubricating oil seeping out from the porous media to achieve self-lubricating and maintenance free operation [8–10]. The porous media unit is employed as an important self-lubricating part for the rolling linear guide [11,12].

The porous oil storage media utilizes pore capillary action to achieve continuous micro output of lubricating oil, and its oil control effect is relatively good [13–15]. The porous media is assembled in the secondary structure of the rolling linear guide, which plays a role in continuously outputting lubricating oil to the lubrication interface and affects the lubrication state during the operation of the rolling linear guide [16,17]. The lubrication performance of the porous oil storage media has an important impact on the operation state of the rolling linear guide [18–20].

The researches indicate that preload degradation [21], nonlinear vibration [22] and nonlinear spring behavior of friction [23] play an important role on the failure Modes [24], wear mechanisms [25] and causes of the rolling linear guide [26,27]. The nonlinear vibration and nonlinear spring are most produced during the operation of rolling linear guide, especially under varying conditions such as speed, load, and mileage; the impact of these factors on the friction and wear mechanisms becomes more significant. These studies are not based on self-lubricating rolling linear guides with porous oil storage media. Nonetheless, their findings indirectly indicate that conditions such as speed, load, and mileage can have an impact on the lubrication of rolling linear guides. However, there is little literature about the influence of porous oil storage media on the lubrication performance during the operation of rolling linear guides [28,29].

Therefore, based on the above analysis, this article fabricates a new type of porous media which is employed in the end cover of the rolling linear guide structures, and the evaluation of lubrication performance of rolling linear guide is systematically evaluated and analyzed. Based on the experiments and tests, this article aims to provide theoretical reference for improving the self-lubricating performance of rolling linear guides.

## 2. Experimental condition

### 2.1 Experimental preparation

Polytetrafluoroethylene (PTFE) powder which produced by Japanese Daikin is employed as base material. The PTFE powder suspension resin with an average particle size of 0.025 mm, an apparent density of 0.48g/cm^3^, a specific gravity of 2.16 (ASTM D4894), a tensile strength of 6240 psi (ASTM D4894), an elongation rate of 400% (ASTM D4894), a shrinkage rate of 0.032 in/in, and a melting temperature of 327°C. The compression permanent deformation rates at 25°C, 100°C, and 200°C were 8.6%, 20%, and 16%, respectively (ASTM D621). Naphthalene (C_10_O_8_) is used as pore forming agent.

The PTFE powder is placed in 120°C dry condition more than 2 hours. The mass ratio of naphthalene to PTFE is 2:8, and they are mixed in pulverize grinder for 5 min and crushed to less than 0.074 mm. The mixture is pressed into a billet with self-preparation mold under a pressure of 3.125MPa. The billet is sintered in a vacuum environment (less than 0.001 Pa) at 385°C for 2 hours. Therefore, the porous media can be obtained.

The mixed billet is completely stored with lubricating oil under vacuum negative pressure. The mass of dry porous media and porous oil storage media after the operation of rolling linear guide are *m*_1_ and *m*_2_, respectively. Thus, the oil storage rate *ψ* is described as:


$$\psi \;=\;\frac{\text{(}m_{\text{2}}\text{-}m_{\text{1}}\text{)}\rho_{\text{1}}}{m_{\text{1}}\rho_{\text{0}}}\times 100$$ (1)

Where, the *ρ*_0_ is the density of experimental lubricant (Mobil DTE 32), *ρ*_1_ is the dry density of unsaturated porous media. The physical characteristic parameters of lubricating oil are shown in Table 1.

**Table 1:** Physical characteristic parameters of lubricating oil.

viscosity grade	kinematic viscosity (cSt)	viscosity index	pour point (°C)	flash point (°C)	density (kg/L)
32	31	102	−18	218	0.85

To analyze the self-lubricating performance of rolling linear guides under different test conditions, the experiment systematically investigated the effects of three operating parameters: slider speed, load, and operating mileage on the self-lubricating performance of the rolling linear guides. The specific values of these three parameters are detailed in Table 2.

**Table 2:** Operating parameters of the self-lubricating experiment for linear rolling guides.

speed (m/s)	load (kN)	operating mileage (km)
0.1	2.938	2
0.4	5.876	4
0.7	8.814	6
1.0	11.752	8
1.3	14.69	10

The value of operating speed increases by 0.3m/s. The rated dynamic operating load *C* of the linear rolling guide (MSA30 type) is 29.38kN. The operating load parameters selected for the test are 0.1*C*, 0.2*C*, 0.3*C*, 0.4*C*, and 0.5*C*, with specific values as shown in the table. The operating mileage of the slider increases by 2 km.

The experiment separately investigates the effects of speed, load, and operating mileage on the self-lubricating performance of rolling linear guides. Experiment parameters indicated with an underline in the Table 2 represent fixed values for that parameter when the other parameters are variable. For example, when operating speed is analyzed as the experimental variable, the values for load and operating mileage are fixed at 2.938kN and 2 km, respectively.

The model of the scanning electron microscope is FEI Phenom prox.

### 2.2 Lubricating oil film thickness test

**Fig 1:**
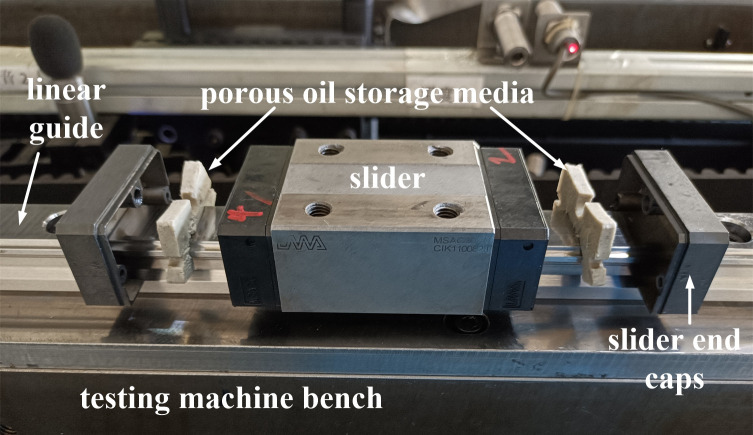
The self-lubricating structure of rolling linear guide.

The porous oil storage media prepared in the experiment is installed between the end of the slider body and the slider end caps, as shown in Fig 1. The lubricating oil stored in the porous media is directly leaked lubricating oil into the friction interface between the slider and the linear guide to achieve self-lubricating of the rolling linear guide.

**Fig 2:**
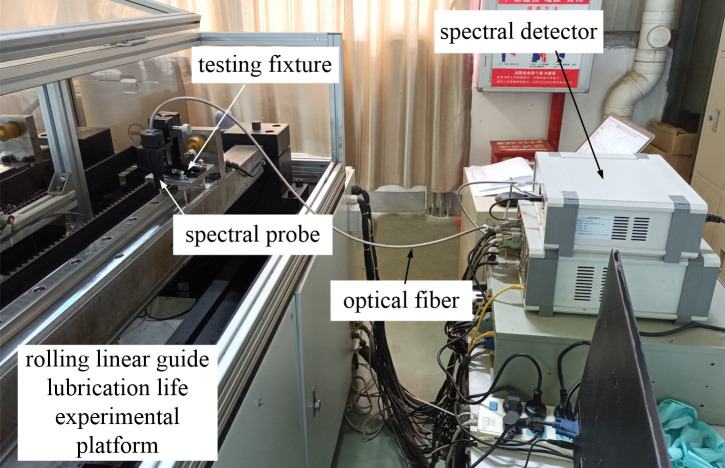
The lubricating oil film thickness testing device for rolling linear guide.

The oil film thickness is a significant indicator for evaluating the self-lubricating performance of rolling linear guides. Spectral interferometry is employed in the oil film thickness test, and the PSD-200W visible light oil film thickness tester from Hangzhou Yangtao Technology Co., Ltd. is used for in situ offline testing of the oil film thickness of rolling linear guides, as shown in Fig 2.

The thickness measurement range of this device is 0.015–0.1 mm, with a refractive index of 100 nm and an accuracy of 2nm or 0.5%. The measurement accuracy is 0.2nm and the measurement speed is less than 20 seconds. The device is equipped with three standard test blocks of different thickness sizes for calibration and testing. The testing equipment adopts the single beam interference method for testing, and the beam is visible light with a wavelength of 380–1000 nm.

The probe fixture can be used to adjust the position of the probe above the guide rail for easy focusing of visible light in the probe. The spectrometer box integrates a spectrometer and a signal acquisition and reception device, mainly used for the emission and feedback signals of the light source. The probe is the main device for visible light emission and reception. The collected signals are processed and analyzed by the computer using Apris Spectra Sys software. The spectrometer is the Ocean Insight A3-SR-100 high-sensitivity spectrometer (380–1000 nm) from the United States, and the tungsten halogen light source (wavelength range 380–2000 nm) is produced by Ocean Insight from the United States. The oil film thickness was measured at 20 different positions on the surface of the guide rail, with three measurements taken at each position to calculate the average value as the oil film thickness for that location.

### 2.3 Principle of optical interference testing for oil film thickness

**Fig 3:**
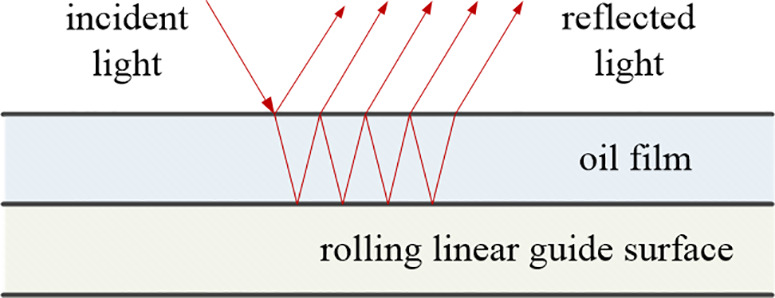
The principle of optical interference measurement for oil film thickness.

When light enters the interior of lubricating oil, multiple reflections occur, and the multiple reflected lights will be enhanced or weakened due to the phase difference between them, which depends on the refractive index and optical path of the lubricating oil as shown in Fig 3. Therefore, the reflection spectrum from lubricating oil is directly related to its thickness. Spectral interferometry is the use of this unique spectral analysis to determine the thickness of a sample.

The results of measuring the oil film with a spectrometer are absolute reflectivity and refractive index, which reflect the percentage of visible light reflected/transmitted from the lubricating oil to the incident light. Therefore, a sample with a known refractive index must be used as a reference. Air can be used as a reference for transmitted light since the transmittance of air is 100%. A silicon wafer can be used as a reference for the reflected light.

The optical properties of materials can be described by two related physical quantities: complex refractive index and complex dielectric constant. The complex refractive index describes the propagation characteristics of light in materials, the real part of the refractive index describes the propagation speed and refractive characteristics of light in different media, and the imaginary part of the refractive index describes the attenuation characteristics of light in media. The optical model of Cauchy Urbach was used in the experiment of measuring oil film thickness, which is characterized as:


$$\;n(\lambda )=A_{n}+\frac{B_{n}}{\lambda^{2}}+\frac{C_{n}}{\lambda^{4}},\;k(\lambda )=A_{k}\exp\left[{1.2398B}_{k}\left(\frac{1}{\lambda }-\frac{1}{C_{k}}\right)\right]$$ (2)

Where, *n*(*λ*) describes the variation of the refractive index of transparent materials in the visible and near-infrared regions as a function of wavelength. The *A*_*n*_ is the constant term of the refractive index, representing the value of the refractive index as the wavelength approaches infinity. The *B*_*n*_ and *C*_*n*_ are the dispersion coefficients, describing the dispersive behaviour of the refractive index as the wavelength decreases. *λ* is the wavelength. *k*(*λ*) describes the exponential decay behaviour of the absorption coefficient of materials near the absorption edge with respect to photon energy. *A*_*k*_ is a normalization constant related to the intrinsic absorption intensity of the material. *B*_*k*_ is a parameter that describes the steepness of the absorption edge, while *C*_*k*_ is the characteristic energy of the absorption edge.

The calculated reflectance/transmittance can be compared with the measured reflectance and transmittance. For known materials, their thickness can be adjusted by computer or manually until the reflection/transmission spectra match the measured results. Use mean squared error (*MSE*) to characterize the difference between the measured reflectance and transmittance curve and the calculated reflectance and transmittance curve.


$$\begin{array}{c} \;MSE=\frac{1}{2N-M}\sum_{k=1}^{N}{\left[\frac{R_{model}\left(\lambda_{k}\right)-R_{\exp}\left(\lambda_{k}\right)}{\sigma (\lambda_{k})}\right]}^{2} \end{array}$$ (3)

*R*_*model*_ and *R*_*exp*_ are the calculated and measured reflectance, *N* is the number of wavelengths, *M* is the number of fitted parameters, and *σ*(*λ*_*k*_) refers to the uncertainty measured at a specific wavelength. The oil film thickness and refractive index of the oil film layer can be fitted automatically by the software. The fitting method in the experiment is Analysis.

## 3. Results

### 3.1 Oil film thickness test

**Fig 4:**
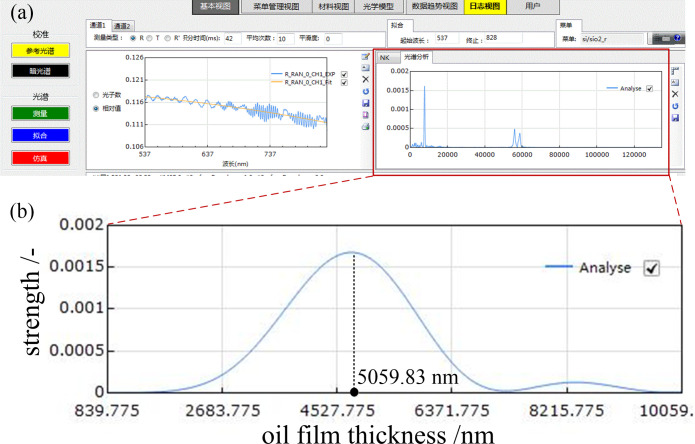
Analysis chart of fitted oil film thickness spectrum test.

According to the principles and experimental conditions established in the above oil film thickness testing analysis, the oil film thickness on the surface of the rolling linear guide can be tested, with the test results shown in Fig 4. For the Fig 4a, the left image represents the reflectance curve, while the right image is the fitted oil film thickness spectral analysis graph, with the horizontal axis representing the oil film thickness values.

The signal tested by the spectrometer can be collected, analyzed, and fitted to obtain a fitted analysis graph, as shown in Fig 4b. The horizontal axis in the figure represents the oil film thickness, and it can be seen that the fitted curve shows a sinusoidal trend. The peak value is the tested oil film thickness value, and the oil film thickness value at the selected test point in this figure is 5059.83 nm.

Therefore, after a certain operating distance of the rolling linear guide, the spectrometer was employed to conduct multi-point oil film thickness testing on the friction surface of the rolling linear guide. Thirty different positions on the surface of the rolling linear guide were selected to test the oil film thickness, and then the average value and variance analysis were taken.

### 3.2 Influence of speed on the oil film thickness

**Fig 5:**
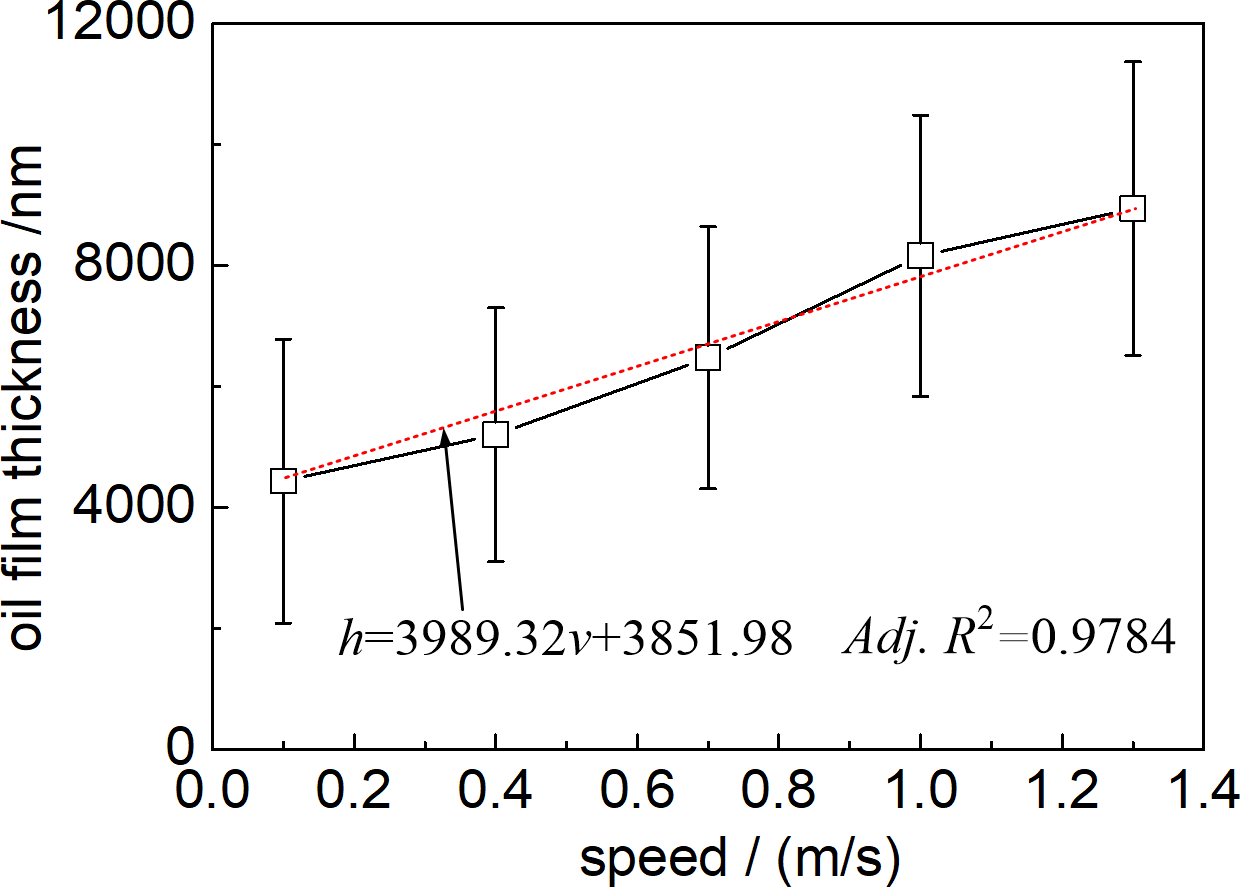
Influence of operating speed of rolling linear guide on the oil film thickness.

It could be observed from Fig 5 that the oil film thickness *h* of the self-lubricating rolling linear guide roughly shows a linear increase trend with the increase of operating speed *v* of rolling linear guide. The change in oil film thickness can be obtained through linear fitting to obtain a linear relationship equation *h* = 3989.32*v* + 3851.98, and the fitted autocorrelation coefficient (*Adj.* R^2^) reaches 0.9784, indicating a good linear fitting relationship.

In addition, the error bar in Fig 5 displays the variation and uncertainty of oil film thickness, with standard deviation values fluctuating between 2098–2425 nm, indicating a large degree of dispersion in oil film thickness. The distribution of oil film thickness on the rolling linear guide surface is relatively uneven, and the distribution of oil film thickness on different friction pair surfaces is relatively discrete. Therefore, the above analysis indicates that the porous media can effectively achieve the seepage of lubricating oil from the friction pair of rolling linear guides, but the lubricating oil leakage rate is not constant and shows a linear increase with the increase of operating speed of rolling linear guide.

### 3.3 Influence of load on the oil film thickness

**Fig 6:**
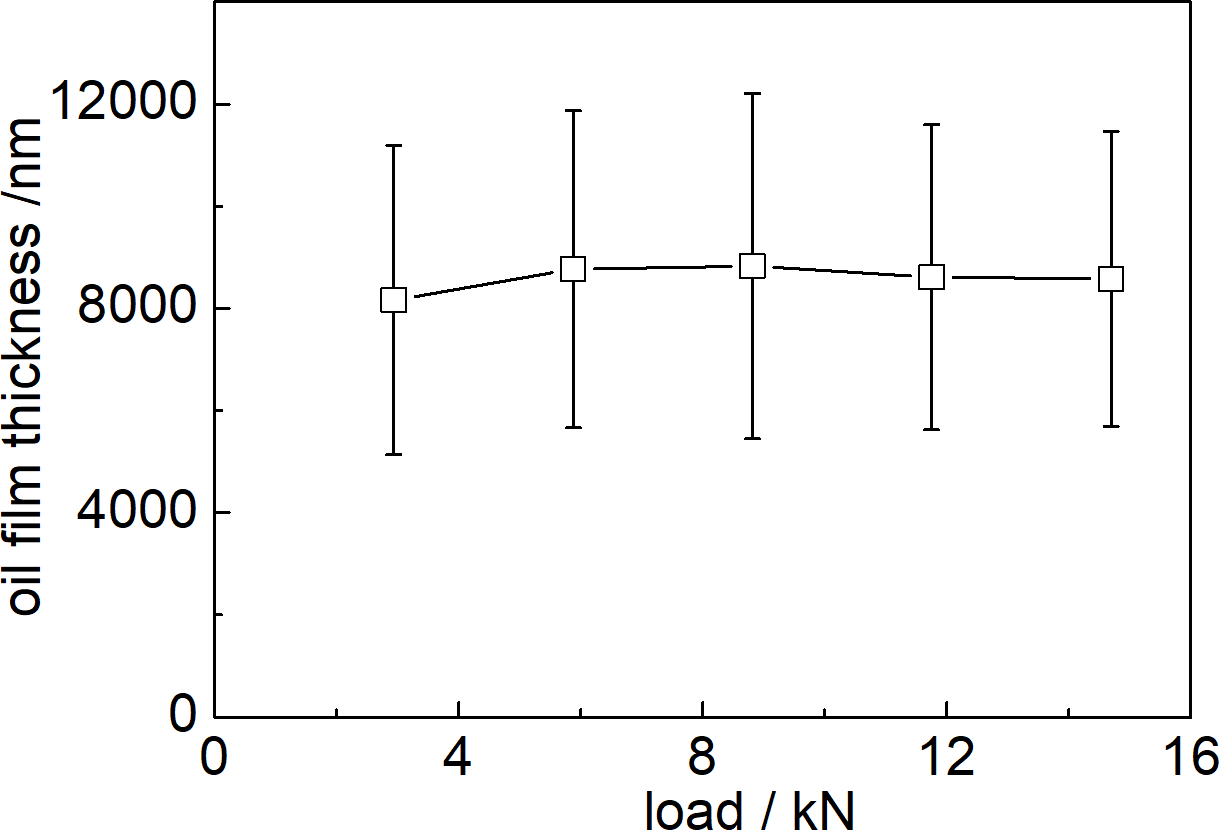
Influence of load of rolling linear guide on the oil film thickness.

The variation trend of the lubricating oil film thickness of the self-lubricating rolling linear guide with load applied on the slider is shown in Fig 6. With the increase of load, the lubricating oil film thickness of the self-lubricating rolling linear guide presents a trend of first gradually increasing and then gradually decreasing, within a small fluctuation value between 8162–8828 nm.

It can be observed that the error bar value of the lubricating oil film thickness is relatively large under different loads, reaching 2886–3382 nm. This also presents that the distribution of oil film thickness on the friction pair surface is relatively discrete, and the large degree of dispersion indicates that the leakage rate of lubricating oil for porous media is relatively irregular. The influence of load on the lubricating oil film thickness of the rolling linear guides is not significant.

### 3.4 Influence of operating mileage on the oil film thickness

**Fig 7:**
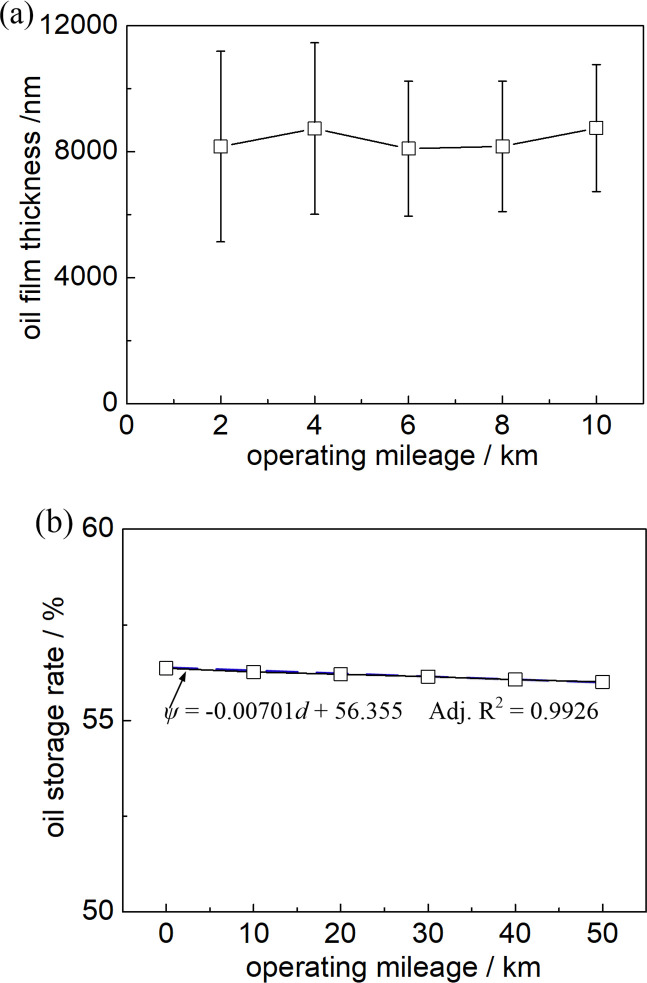
Influence of operating mileage of rolling linear guide on the oil film thickness (a) and oil storage (b).

The lubrication performance characteristics of self-lubricating rolling linear guides at different operate ranges are shown in Fig 7. The thickness of the oil film on the self-lubricating rolling linear guide displays a trend of first increasing (Fig 7a), then decreasing, and then increasing with the increase of operating mileage. The amplitude of the change is not large, and the fluctuation range is between 8094nm and 8748nm. In addition, the error bar value shows that the distribution of lubricating oil film thickness is relatively discrete.

It can be obtained by tracking and analyzing the changes in oil storage rate of the rolling linear guide during operation from Fig 7b. As shown in the figure, with the increase of operating mileage, the oil storage rate shows a slow linear decreasing trend. The linear fitting relationship between the oil storage rate *ψ* and operating mileage *d* of the self-lubricating rolling linear guide is *ψ* = −0.000701*d* + 56.355, and the fitted autocorrelation coefficients (*Adj*. R^2^) reach 0.9926. That shows a good linear fitting relationship.

## 4. Discussion

### 4.1 Minimum oil film thickness for self-lubricating rolling linear guide

The experimental rolling linear guide is MSA30 type, ant its contact state is point contact. Among various films thickness calculation formulas for isothermal point contact elastohydrodynamic lubrication problems, the most commonly used is the Hamrock-Dowson formula. It is suitable for situations where the direction of the suction speed coincides with the short half axis of the contact ellipse, which is consistent with the transmission of the rolling linear guide pair. The Hamrock-Dowson formula can be expressed as [19]:


$$\;H=\frac{h_{\min}}{R_{x}}=\frac{0.363G^{0.49}U^{0.68}(1-e^{-0.68k})}{W^{0.073}}$$ (4)

In the formula, *h*_min_ is the minimum oil film thickness, *U* is a dimensionless velocity parameter, *G* is a dimensionless material parameter, and *W* is a dimensionless load parameter, *k* is ellipticity, *k* = 1.03(*R*_*y*_/*R*_*x*_)^0.64^, *R*_*x*_ and *R*_*y*_ are the equivalent curvature radius of the two surfaces in the *xoz* plane and *yoz* plane, respectively. The specific description is:


$$\frac{1}{R_{x}}=\frac{1}{R_{1x}}+\frac{1}{R_{2x}}$$ (5)

$$\frac{1}{R_{y}}=\frac{1}{R_{1y}}+\frac{1}{R_{2y}}$$ (6)

*R*_1x_ and *R*_2*x*_ are the curvature radii of two surfaces in the *xoz* plane, while *R*_1*y*_ and *R*_2y_ are the curvature radii of two surfaces in the *yoz* plane. There are three dimensionless parameters:

(1) Material parameter *G *= *αE*, *E* is the modulus of elasticity, where:


$$\begin{array}{c} \frac{1}{E}=\frac{1}{2}\left(\frac{1-\gamma_{1}^{2}}{E_{1}}+\frac{1-\gamma_{2}^{2}}{E_{2}}\right) \end{array}$$ (7)

In the equation, *γ*_1_ and *γ*_2_ are Poisson’s ratios. *E*_1_ and *E*_2_ are modulus of elasticity for material 1 and 2 of the friction pairs.

(2) The speed parameter *U* is represented as:


$$\;U=\frac{\eta_{0}U^{\prime }}{ER_{x}}$$ (8)

In the formula, *U*’ = 0.5(*U*_1_ + *U*_2_) is the average surface velocity, and *U*_1_ and *U*_2_ are the tangential velocities at the contact points of the two surfaces, respectively.

(3) The load parameter *W* is represented as:


$$\;W=\frac{W^{\prime }}{ER_{x}^{2}}$$ (9)

Where, the *W*’ is the load that is received. The lubrication state of the rolling linear guide depends not only on the thickness of the oil film, but also on the surface roughness of the friction pair. The lubrication state of the rolling linear guide can be characterized by the oil film parameter *Λ* [20]:


$$\;\Lambda =\frac{h_{\min}}{\sqrt{\sigma_{1}^{2}+\sigma_{2}^{2}}}$$ (10)

Where, the *σ*_1_ and *σ*_2_ are the root mean square deviation for the surface roughness of the two friction pairs of the linear guide and the steel ball, respectively (*σ* = 1.25*Ra*). Therefore, according to the theory of elastohydrodynamic lubrication, the lubrication state of rolling linear guides can be characterized and analyzed by oil film parameters:

(1) When the oil film parameter *Λ* > 3, the friction pair is in a fully film elastohydrodynamic lubrication state, with no micro convex contact and good lubrication.
(2) When the oil film parameter 1 < *Λ *≤ 3, the friction pair is in a partially film elastohydrodynamic lubrication state, with micro convex contact and certain wear, and normal lubrication.
(3) When the oil film parameter *Λ *≤ 1, the friction pair is in a boundary lubrication state, with severe wear and poor lubrication.

The steel ball used in the experimental rolling linear guide is manufactured by Jiangsu Lixing General Steel Ball Co., Ltd. The diameter of the steel ball is 4.7625 mm, the material is GCr15. The standardized surface roughness value of the steel ball used in the slider is 0.020 μm, and the surface roughness of the linear guide after precision grinding is 0.3 μm. By substituting the surface roughness value into the calculation formula for the minimum oil film thickness mentioned above, it can be concluded that for the experimental rolling linear guide, the minimum oil film thickness *h*_min_ is:

(1) When *h*_min_ > 1.128 μm, the friction pair is in a fully film elastohydrodynamic lubrication state with good lubrication.
(2) When 0.376 μm < *h*_min_ ≤ 1.128 μm, the friction pair is in the state of partially elastohydrodynamic lubrication.
(3) When *h*_min_ ≤ 0.376 μm, the friction pair is in a boundary lubrication state and presents severe wear.

Combined with Fig 5, it can be seen that the thickness of the lubricating oil film is 4437.77μm, 5203.08μm, 6477.03μm and 6477.03μm respectively under different speed conditions. 8162.73 microns and 8941.92 microns, the oil film thickness is greater than 1.128μm, it can be seen that under the self-lubricating action of the porous oil storage media prepared by the experiment, the rolling linear guide can realize the whole film elastic flow lubrication, which is mainly due to the increase of speed, the parallel contact area of the oil film is continuously shortened, the necking phenomenon of the oil film becomes weaker [19], and the increase of operating speed also accelerates the fluidity of the lubricating oil in the porous oil storage media, which is beneficial to the lubrication state of the contact area of the friction pair.

According to Fig 6, the thicknesses of the lubricant film under different speed conditions are 8162.73μm, 8767.95μm, 8828.51μm, 8611.11μm, and 8579.22μm, all of which are greater than 1.128μm. The rolling linear guide rails remain in a full film elastohydrodynamic lubrication state under various loads. The increase in load does not significantly affect the oil film thickness. This is primarily because, although the increase in load may cause a necking phenomenon in the contact area of the friction pair, the load does not directly act on the surface of the porous oil reservoir media [30]. The characteristics of the porous oil reservoir media, which allows lubricant to seep to the surface of the friction pair via capillary action, remain unaffected, thus enabling a continuous supply of lubricant to the friction pair surface for adequate lubrication.

It can be observed from Fig 7 that the oil film thickness at different operating mileages is 8162.73μm 8731.62μm, 8094.83μm, 8167.52μm, and 8748.71μm, respectively. The oil film thickness is consistently greater than 1.128μm, indicating that the rolling linear guide is operating under a full film hydrodynamic lubrication state at different mileage [19,20]. The increase in mileage does not have a significant impact on the oil film thickness, primarily due to the fact that within the operating mileage range of the experimental conditions (50 km), the porous oil storage media is still able to effectively permeate sufficient lubricating oil to ensure full film hydrodynamic lubrication of the rolling linear guide friction pair. Therefore, the influence of mileage on oil film thickness is not significant under the experimental conditions.

### 4.2 Surface morphology characteristics of porous media

**Fig 8:**
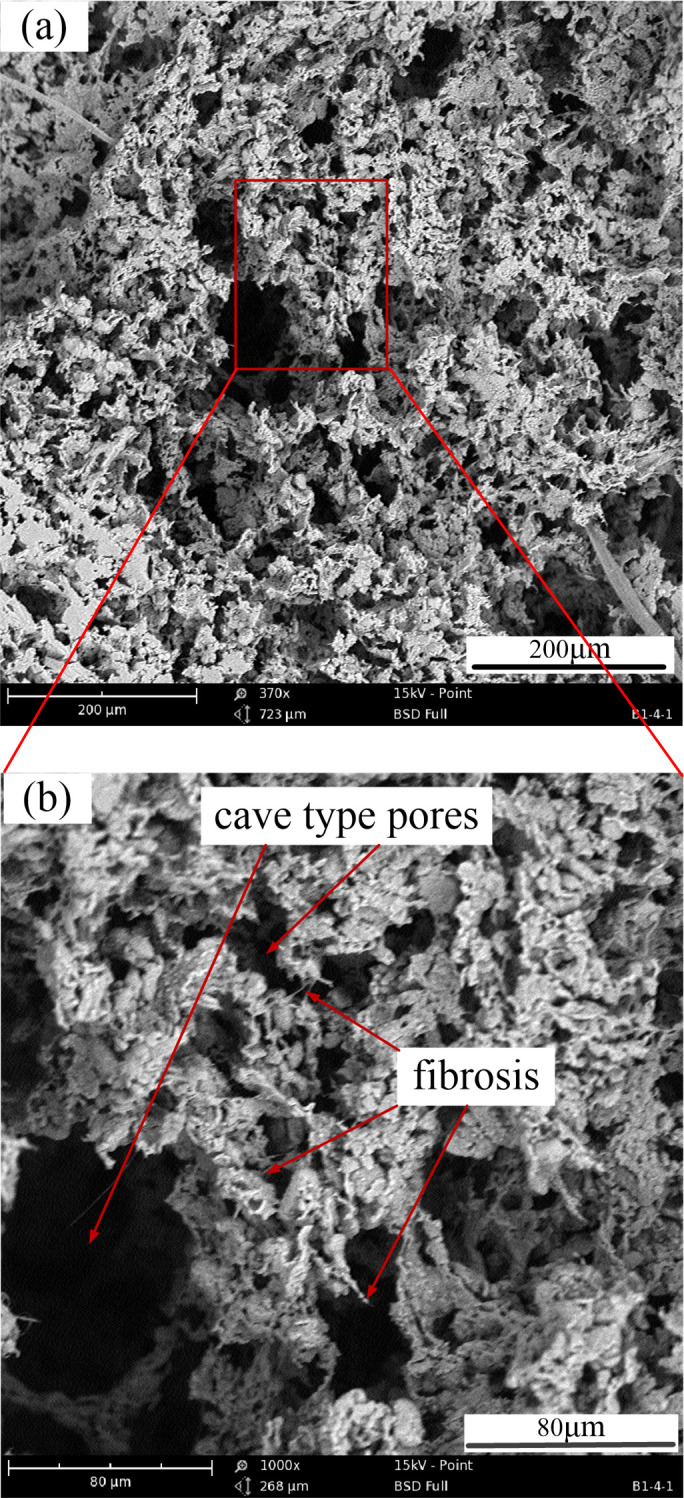
Surface micromorphology (a) and localized magnified microstructure (b) of porous media.

The internal morphology of porous media is shown in Fig 8. There are a large number of interconnected large gaps inside the porous media (Fig 8a), and each pore penetrates inward to form a cave type shape. The formation and structure of the pores are conducive to increase the storage capacity of lubricating oil in the porous media, and achieve continuous output of lubricating oil to the friction pair inter-face during the operation of the rolling linear guide.

The microstructure of the localized magnified pore structure in porous media is shown in Fig 8b. The internal pore structure distribution of porous media is relatively uniform, and the connectivity between pores is good. The interconnected pores can form effective pore channels for the storage and circulation of lubricating oil. More cavernous pore structures and obvious fibrous tissue structure are formed in the porous media. Fibrotic tissue structure is beneficial for porous media to lock lubricating oil within the molecular tension range, thereby achieving permeation and oil control of lubricating oil.

Based on the lubrication structure of the slider (Fig 1), experimental results (Figs 5–7), and microscopic image analysis (Fig 8), it can be concluded that the reciprocating acceleration and deceleration motion of the slider within a limited range results in an increase in the flow velocity of lubricating oil in the porous media, causing more lubricating oil to seep out towards the friction surface and increase the thickness of the lubricating oil film. Therefore, as the operating speed of the slider increases, the thickness of the oil film also increases.

Secondly, the load directly acts on the metal shell of the slider, and the increase in load does not directly act on the surface of the porous media, which cannot generate seepage pressure. Therefore, the change in load has no significant effect on the thickness of the oil film.

Furthermore, since the porous media can continuously leak lubricating oil to lubricate the friction surface during its lubrication life (Fig 8a), the change in oil film thickness is not significant. As the amount of lubricating oil seeping out gradually increases, the amount of lubricating oil stored inside the porous media decreases, resulting in a decrease in its oil storage rate (Fig 8b).

## 5. Conclusions

1) The influence of operating speed on the oil film thickness on rolling linear guides is significant, and the oil film thickness is linearly proportional to the operating speed of the slider. The influence of operating load and operating mileage on the oil film thickness is not significant. The oil storage rate gradually decreases with the operating mileage increases.
2) The internal pores of porous media which are interconnected with a fibrous tissue structure of cave type present good connectivity, and have different shapes and sizes. The interconnection of various pores is beneficial for oil storage, oil control, and oil leakage during the operation of rolling linear guides.
3) The self-lubricating rolling linear guide prepared is in a fully film elastohydrodynamic lubrication state during different experimental conditions, which can achieve good lubrication of the friction pair of the rolling linear guide.

## Supporting information

S1 Dataset experiment data. (XLS)

S2 Dataset figure data. (XLSX)

S3 Dataset image analysis data. (XLSX)
